# Ammonium Formate-Pd/C as a New Reducing System for 1,2,4-Oxadiazoles. Synthesis of Guanidine Derivatives and Reductive Rearrangement to Quinazolin-4-Ones with Potential Anti-Diabetic Activity

**DOI:** 10.3390/ijms222212301

**Published:** 2021-11-14

**Authors:** Paola Marzullo, Sonya Vasto, Silvestre Buscemi, Andrea Pace, Domenico Nuzzo, Antonio Palumbo Piccionello

**Affiliations:** 1 Dipartimento di Scienze e Tecnologie Biologiche, Chimiche e Farmaceutiche-STEBICEF, Università degli Studi di Palermo, 90128 Palermo, Italy; paola.marzullo@unipa.it (P.M.); silvestre.buscemi@unipa.it (S.B.); andrea.pace@unipa.it (A.P.); domenico.nuzzo@cnr.it (D.N.); 2 Euro-Mediterranean Institute of Science and Technology (IEMEST), 90139 Palermo, Italy; 3 Consiglio Nazionale delle Ricerche, Istituto di Biofisica (CNR-IBF), 90146 Palermo, Italy

**Keywords:** 1,2,4-oxadiazole, reduction, quinazolin-4-one, acylguanidine, diacylguanidine, ammonium formate, palladium

## Abstract

1,2,4-Oxadiazole is a heterocycle with wide reactivity and many useful applications. The reactive O-N bond is usually reduced using molecular hydrogen to obtain amidine derivatives. NH_4_CO_2_H-Pd/C is here demonstrated as a new system for the O-N reduction, allowing us to obtain differently substituted acylamidine, acylguanidine and diacylguanidine derivatives. The proposed system is also effective for the achievement of a reductive rearrangement of 5-(2′-aminophenyl)-1,2,4-oxadiazoles into 1-alkylquinazolin-4(1*H*)-ones. The alkaloid glycosine was also obtained with this method. The obtained compounds were preliminarily tested for their biological activity in terms of their cytotoxicity, induced oxidative stress, α-glucosidase and DPP4 inhibition, showing potential application as anti-diabetics.

## 1. Introduction

1,2,4-Oxadiazoles are five-membered heterocycles that have been widely studied for their valuable applications ranging from the pharmaceutical field to material science [1]. The use of a 1,2,4-oxadiazole ring as an isosteric replacement of esters and amides increased their presence in medicinal chemistry projects [2] with biological applications ranging from the treatment of nonsense mutations [3,4] and Alzheimer’s disease (AD) [5,6,7] to antibiotics [8,9,10] and antitumorals [11,12]. Applications in the field of materials chemistry, such as liquid crystals [13] metal sensors [14], OLED [15], energetic materials [16] and gas sorbing/releasing systems [17,18,19], have been reported. The high tendency to react and rearrange of this low-aromatic ring is one of the features that is mainly related to O–N bond breaking [20]. 

In this field, the reduction of the O–N bond is usually performed with classical methods, such as catalytic hydrogenation or Fe/AcOH catalyzed reduction with the initial formation of intermediate acylamidines that hydrolyze in situ to give amidines [21,22]. Notably, the ammonium formate-Pd/C system, despite its widespread use [23] was never employed as a 1,2,4-oxadiazole O–N bond reducing agent. In this work, we report the optimization of the reduction process of 1,2,4-oxadiazoles to directly obtain amidine and guanidine as well as quinazolin-4-ones through a reduction-induced heterocyclic rearrangement. All these compounds are of great interest due to their potential biological activity [24,25,26,27,28,29,30,31,32,33,34].

## 2. Results

### 2.1. Reduction Reactions

**Scheme 1:**
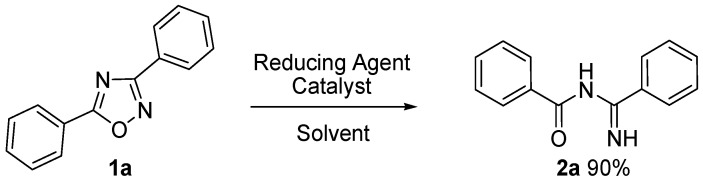
Reduction of 3,5-diphenyl-1,2,4-oxadiazole **1a**.

We used 3,5-diphenyl-oxadiazole **1a** as a model substrate in the optimization of the reaction conditions (Scheme 1). 

As shown in Table 1, the corresponding acylamidine **2a** was obtained only in the presence of polar protic solvents (entries 8–11); however, the desired product was obtained in good yield and in a shorter time by heating the reaction in methanol (entry 12). Interestingly, formic acid could also be used as a reducing agent (entry 10–11). The use of zinc dust or the absence of catalyst did not produce the desired product (entry 6–7).

**Scheme 2:**
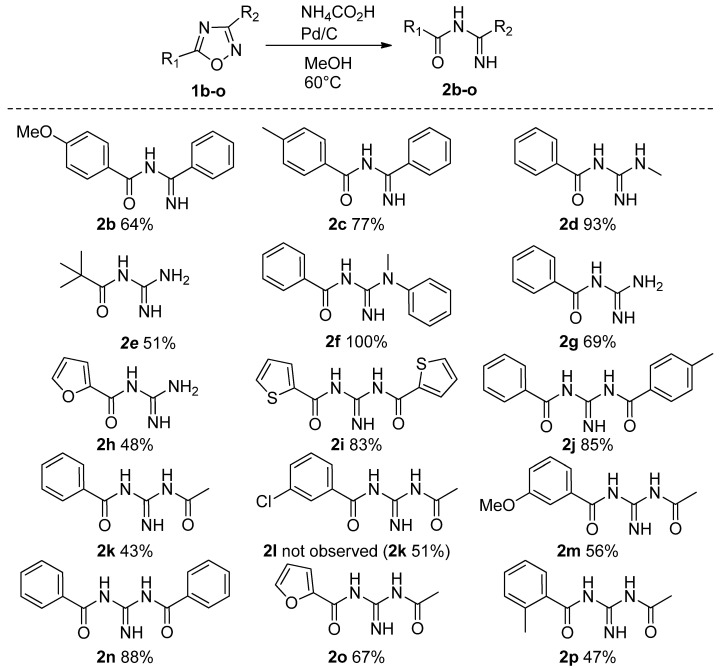
Reduction of 1,2,4-oxadiazoles **1** to amidine and guanidine derivatives **2**.

**Scheme 3:**
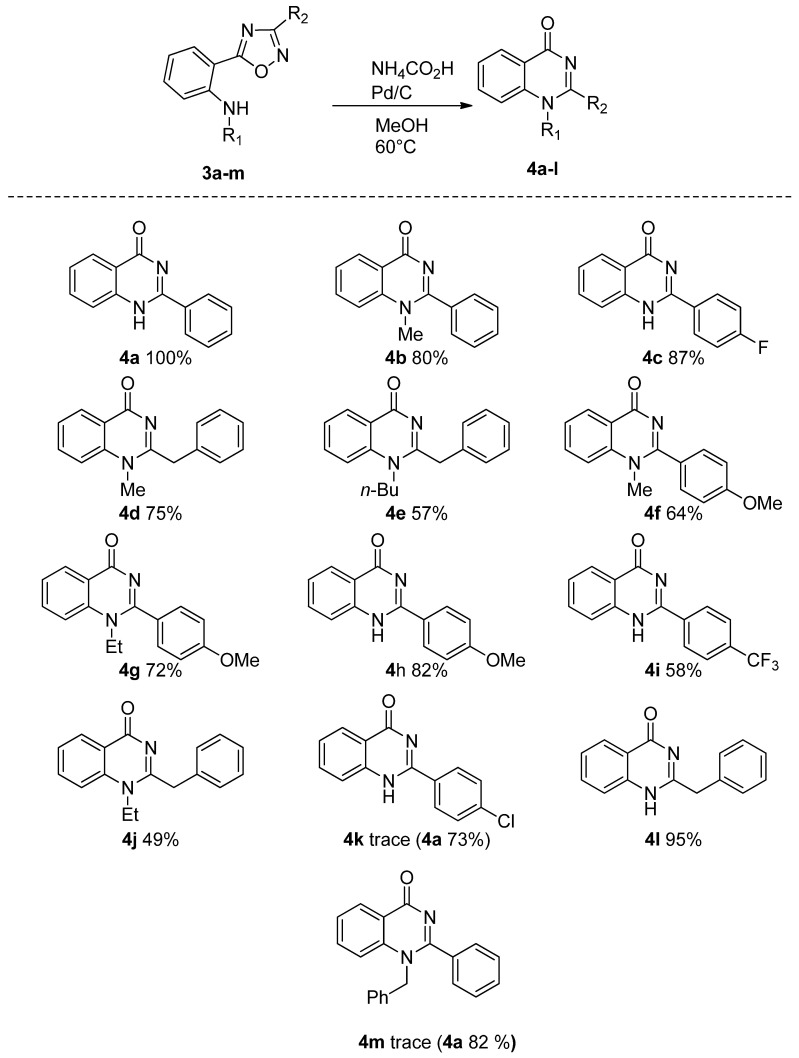
Reduction of 1,2,4-oxadiazoles **3** to quinazolin-4-ones **4**.

The scope and limitations of these reduction reactions under optimized conditions using NH_4_CO_2_H and Pd/C (5%) in MeOH at 60 °C, were investigated with variously substituted oxadiazoles **1b-o** (Scheme 2) and **3a-m** (Scheme 3). Acylamidine, acylguanidine and diacylguanidine derivatives **2b-o** were obtained in good to excellent yields with the only exception of chlorinated compound **2l,** which, in addition to the reduction of the O–N bond, gave the corresponding de-halogenated product **2k** (Scheme 2). 

Furthermore, the reaction of ortho-aminophenyl-1,2,4-oxadiazoles **3** was considered as a potential reductive ring-rearrangement that allowed us to obtain the corresponding quinazolin-4-ones **4** in good to excellent yields (Scheme 3). The observed limitations of the proposed reaction are referred to the simultaneous dechlorination of compound **4k** to compound **4a** and by the reductive debenzylation process of **3m** to compound **4a**. The reaction performed on *N*-alkyl substituted oxadiazoles **3b**,**d**–**g**,**j** provided the regioselective formation of *N*(1) substituted quinazolinones **4**.

**Scheme 4:**
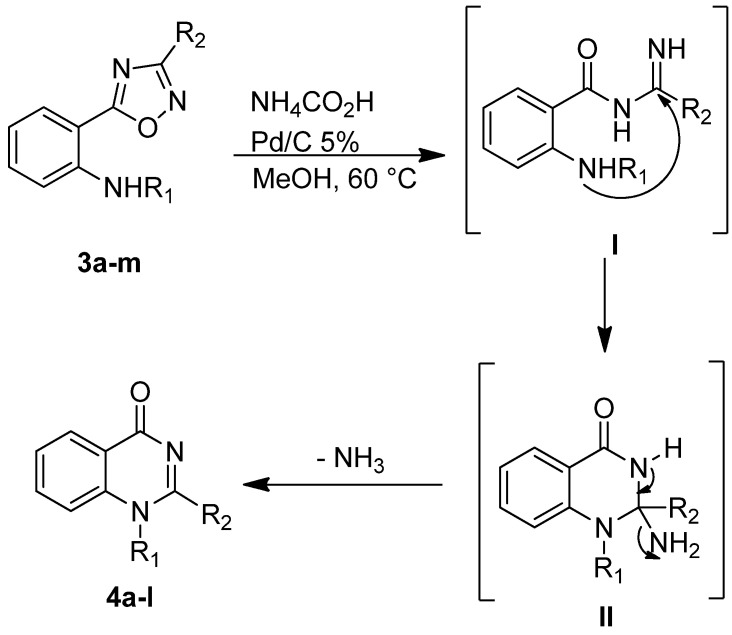
Plausible mechanism for the reductive rearrangement of 1,2,4-oxadiazoles **3** into quinazolin-4-ones **4**.

From a mechanistic point of view, the reductive rearrangement of oxadiazoles **3** into quinazolin-4-ones **4** could be rationalized with an initial reduction of the O–N bond, to amidine **I** accompanied by an intramolecular cyclization into intermediate **II**, followed by the elimination of ammonia, to obtain compound **4** (Scheme 4) [35].

### 2.2. Inhibition Test of Dipeptidyl-Peptidase IV (DPPIV) Enzyme

**Figure 1:**
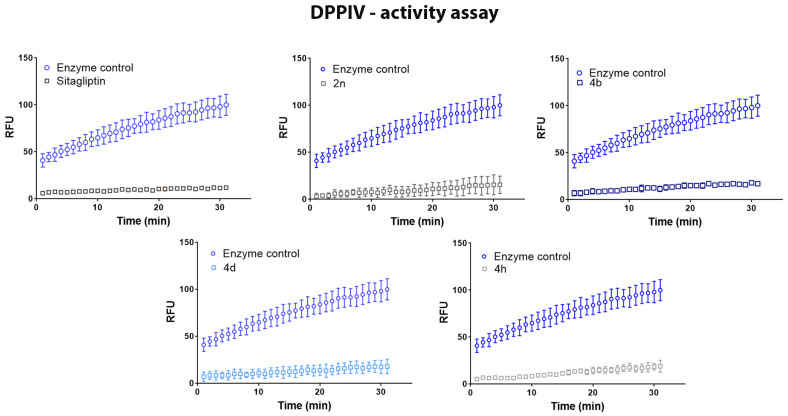
Representative curves of the dipeptidyl peptidase activity in the presence of the compounds (100 µM). Sitagliptin was used as positive control for the test. Fluorescence values (RFU) were measured in kinetic mode. Data were analyzed through linear regression analysis performed for the initial points of the curve.

The synthesized compounds were screened for their in vitro DPPIV inhibitor activity. The DPPIV enzyme is a therapeutic target in diabetic disease, and its inhibition increases the glucagon-like peptide levels with a consequent decrease of postprandial glycemia [36]. DPPIV activity was determined by cleaving the substrate to yield a fluorescent product (λ_ex_ = 360/λ_em_ = 460), proportional to the enzymatic activity. As shown in Figure 1, the tested compounds showed an important inhibitor activity at 100 µM, in particular, the alkaloid Glicosine **4d** [37] and its derivatives. 

The guanidine derivatives **2k**, **2n** and **2e** inhibit the enzyme at the same concentration (Table 2). The other tested compounds were not active or gave spectral interference with the assay. For those compounds with a good inhibitor activity (>83%), the IC_50_ was determined. Table 2 shows the IC_50_ values for the most active compounds, revealing a good potency in the sub-micromolar range for all compounds and an IC_50_ value for Glicosine of the same order of that of reference drug Sitagliptin. 

### 2.3. Inhibition Test of α-Glucosidase Enzyme

The α-glucosidase enzyme catalyzes the hydrolysis of various carbohydrate substrates into monosaccharides that are easily absorbed through the small intestine [37]. The inhibition of α-glucosidase slows down the glycemic levels and represents a therapeutic strategy for diabetic patients. Compounds were evaluated in vitro for their alpha-glucosidase inhibitory activity. The assay is based on enzyme catalyzed hydrolysis of 4-nitrophenyl β-D-glucopyranoside (pNPG) to 4-nitrophenol measured for absorbance at 490 nm. The inhibitor Acarbose was used as a positive control. The tested compounds gave absorbance values similar to that of the uninhibited enzyme (data not shown); therefore, no inhibitory activity was envisaged.

### 2.4. Cytotoxicity Assay and Evaluation of Intracellular Redox State

**Figure 2:**
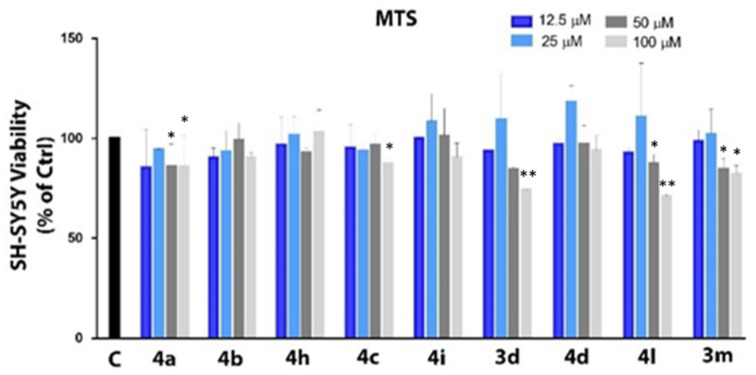
Viability percentage of SH-SY5Y cells exposed for 24 h to serial concentrations of the compounds. Data were compared by one-way ANOVA followed by Dunnett’s multiple-comparison test: *, *p* < 0.05; **, *p* < 0.01 as compared to control (C) group.

The toxicity of several derivatives was assessed on a human neuroblastoma cell line SH-SY5Y (ATCC: CRL–2266^TM^) and on human adenocarcinoma alveolar basal epithelial cells A549 (CCL–185) (results not shown). Cells were exposed for 24 h to four concentrations of the compounds. The absorbance values measured in the MTS assay were proportional to the cell viability. The results were similar for both cell lines. At the lower concentrations of 12.5 and 25 µM, the compounds showed a good viability percentage comparable with that of untreated cells (Figure 2). Some compounds exerted a toxic effect at the concentrations of 50 and 100 µM. The observation of the cell morphology under the electron microscope showed crystals formation for the compounds **4i** and **3m**.

The assay with 2′,7′-dichlorofluorescin-diacetate (DCFH-DA) was performed to evaluate the effect of the compounds on the intracellular redox potential of SH-SY5Y cells and A549 cells (results not shown). DCFH-DA is a non-fluorescent and cell-permeant molecule used as an indicator for reactive oxygen species (ROS). Inside the cell, the DCFH-DA is hydrolyzed by intracellular esterase with cleavage of the acetate group. 

**Figure 3:**
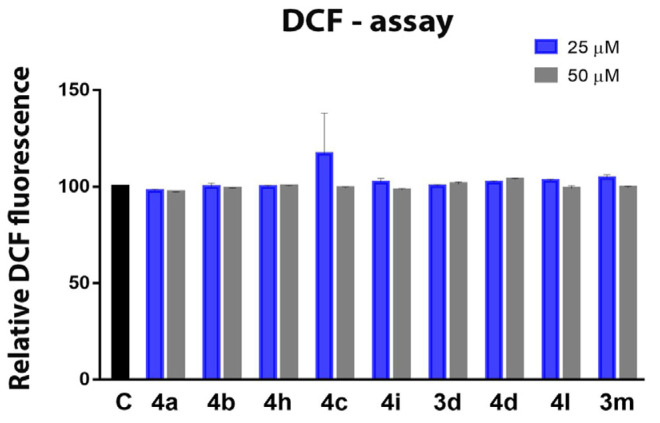
Results of the DCF assay performed on SH-SY5Y treated with representative compounds at 25 and 50 µM.

In the presence of ROS, the molecule is oxidized to the highly fluorescent 2′,7′-dichlorofluorescin (DCF). The emitted fluorescence is proportional to the quantity of the intracellular ROS. The treated cells gave fluorescence values similar to the untreated cells (C) (Figure 3). This result suggests that the tested compounds do not stimulate ROS production and, therefore, do not give oxidative stress.

## 3. Discussion

The reduction reactions are one of the most investigated transformations in organic synthesis, and there is a growing interest to identify new reagents and methodologies for that purpose. Here, we report a new reduction method of 1,2,4-oxadiazole involving readily available and cheap ammonium formate as a hydrogen source and Pd/C as catalyst. This method allowed us to obtain, using mild conditions, acylamidine, acylguanidine and diacylguanidine without hydrolysis to amidines. The reduction of 5-(2′-aminophenyl)-1,2,4-oxadiazoles gave a reductive rearrangement into 1-alkylquinazolin-4(1*H*)-ones. 

Particularly, the reduction of O-N bond gave the *N*-acylamidines that cyclize in situ to produce quinazolin-4-ones in excellent yields. Usually, quinazolinone compounds can be synthetized by oxidative cyclization of benzamide or benzonitrile [38,39]. Our method, compared to the classical one, does not involve an oxidizing agent and it allowed to obtain quinazolinone derivatives alkylated on nitrogen at position 1. This feature is of interest considering that the alkylation of quinazolinones usually occurs at N(3) or gives mixtures of different alkylation products. 

Considering the assessed hypoglycemic activity of Glicosine analogues of the hypoglycemic alkaloid glycosine [37] and the similar activity of similar compounds, we decided to test the potential application of synthesized compounds as antidiabetic drugs. Considering that the mode of action of Glicosine is still unknown, we tested major target of drugs with hypoglycemic activity against DPPIV and α-glucosidase. The obtained compounds were inactive against α-glucosidase; however, four quinazolinones **4b**,**d**,**e**,**l** were able to inhibit DPPIV in the sub-micromolar range. 

These results confirm previously reported docking studies that proposed the DPPIV enzyme as a biological target for Glicosine [37]. Considering recent evidence regarding the pro-oxidant and neurotoxic activity of hypoglycemic drug Metformin [40], we also tested the pro-oxidant activity of selected compounds. As shown by MTS and dichlorofluorescein assays, the derivatives have a good cytocompatibility in terms of cell viability and pro-oxidant activity. 

## 4. Materials and Methods

### 4.1. Materials

All solvent and reagents were obtained from commercial sources and were used without purification. The reactions were monitored by thin layer chromatography (TLC). The synthesized compounds were purified by silica flash chromatography using silica gel (0.040–0.063 mm) and a mixture of ethyl acetate and petroleum ether (fraction boiling in the range of 40–60 °C) in various ratios as the eluent. The melting points were determined on a Kofler apparatus. FTIR spectra (Nujol or CH_2_Cl_2_) were determined with a Cary 630, Agilent Technologies instrumentation (Agilent Technologies, Santa Clara, CA, USA). ^1^H-NMR, ^13^C-NMR and HPLC/MS were utilized to verify the structure and purity of synthesized compounds.

^1^H-NMR and ^13^C-NMR were recorded at 300 MHz for ^1^H and 62.5 MHz for ^13^C; DMSO-*d*_6_ or CDCl_3_ were used as solvent and TMS as an internal standard. HRMS spectra were recorded in positive or negative mode with HPLC/MS (6540 UHD Accurate Mass Q-TOF LC/MS—Agilent Technologies, Santa Clara, CA, USA) and Dual AJS ESI source. The compounds **1a** [41], **1b [42]**, **1c** [43], **1d** [44], **1e**,**j**,**o** [45], **1f** [46], **1g**,**h** [47], **1i**,**k**,**n** [48], **1l**,**m** [49] and **1p [50]** were obtained as previously reported. The enzyme α-glucosidase Type I from *Saccharomyces cerevisiae*, acarbose and 4-nitrophenil-α-d-glucopyranoside have been bought by Sigma Aldrich. DPPIV Activity Fluorometric Assay Kit (Sigma Aldrich, St. Louis, MO, USA) was used for inhibition test of dipeptidyl peptidase. Both inhibition assays were conducted in 96-well plates using a 96-well microplate reader (BioTek, Winooski, VT, USA)

### 4.2. General Procedure for the Synthesis of N-Acylamidines, Quinazolin-4-(1H)-One, Acyl Guanidine and Diacyl Guanidine

To a solution of appropriate oxadiazole (1 mmol) in MeOH (20 mL), NH_4_CO_2_H (3 mmol) was added as a reducing agent and Pd/C 5% (10 mg) was added as a catalyst. The reaction mixture was heated to 60 °C for 1 h (monitored with TLC). The solution was filtered to remove palladium and the filtrate was evaporated under vacuum. The residue was treated with water and extracted with ethyl acetate. If necessary, the product was further purified by column chromatography using petroleum ether and ethyl acetate (1:1) as eluent. 

*N-[Imino(phenyl)methyl]benzamide***2a**. White solid; yield 90%; m.p. 103–104 °C; ^1^H-NMR (300 MHz, DMSO-*d*_6_) δ ppm 7.36–7.45 (m, 6H), 8.14–8.28 (m, 4H). Spectroscopic data are consistent with the literature [43].

*N-[Imino(phenyl)methyl]-4-methoxybenzamide***2b**. White solid; yield 64%; m.p. 98–101 °C; ^1^H-NMR (300 MHz, DMSO-*d*_6_) δ ppm 3.86 (s, 3H), 7.10 (d, *J* = 8.9 Hz, 2H), 7.46–7.57 (m, 3H), 8.20–8.27 (m, 4H) [43].

*N-[Imino(phenyl)methyl]-4-methylbenzamide***2c**. White solid; yield 77%; m.p. 100–102 °C; ^1^H-NMR (300 MHz, CDCl_3_) δ ppm 2.41 (s, 3H), 7.24–7.29 (m, 2H), 7.51–7.61 (m, 3H), 7.70 (d, 2H, *J* = 8.1 Hz), 8.07 (d, *J* = 6.9 Hz, 2H), 8.30 (d, *J* = 8.1 Hz, 2H). Spectroscopic data are consistent with the literature [51]. 

*N*-*(N-Methylcarbamimidoyl)benzamide***2d**. White solid; yield 93%; m.p. 157–159 °C; ^1^H-NMR (300 MHz, DMSO-*d*_6_) δ ppm 2.79 (s, 3H), 6.61 (s, 2H), 7.35–7.43 (m, 3H), 8.08 (s, 2H), 8.65 (s, 1H); ^13^C-NMR (62.5 MHz, DMSO-*d*_6_) δ ppm 27.8, 128.1, 128.9, 130.8, 139.7, 140.5, 175.5; HRMS (ESI) [M − H]^−^ calculated for C_9_H_11_N_3_O: 176.0829 found: 176.0829.

*N-Carbamimidoylpivalamide***2e**. Yellow solid; yield 51%; m.p. 168–170 °C; FTIR (Nujol) ν: 3403, 3182, 1696, 1595, 767 cm^−1^; ^1^H-NMR (300 MHz, CDCl_3_) δ ppm 1.30 (s, 9H), 8.65 (bs, 4H); ^13^C-NMR (62.5 MHz, CDCl_3_) δ ppm 26.5, 40.9, 157.3, 181.7; HRMS (ESI) [M + H]^+^ calculated for C_6_H_13_N_3_O: 144.1131 found: 144.1131.

*N-(N-Methyl-N-phenylcarbamimidoyl)benzamide***2f**. White solid; yield 100%; m.p. 99–101 °C; ^1^H-NMR (300 MHz, DMSO-*d*_6_) δ ppm 3.43 (s, 3H), 7.35–7.53 (m, 8H, overlap), 8.02–8.04 (m, 2H); ^13^C-NMR (62.5 MHz, DMSO-*d*_6_) δ ppm 30.2, 127.7, 127.8, 128.2, 129.1, 130.0, 131.3, 139.4, 143.2, 160.8, 175.6; HRMS (ESI) [M + H]^+^ calculated for C_15_H_15_N_3_O: 254.1288 found: 254.1302.

*N-Carbamimidoylbenzamide***2g**. White solid; yield 69%; m.p. 153–155 °C; ^1^H-NMR (300 MHz, CDCl_3_) δ ppm 3.94 (s, 1H), 7.40–7.53 (m, 3H), 8.06 (d, *J* = 8.7 Hz, 1H), 8.16 (d, *J* = 7.5 Hz, 2H). Spectroscopic data are consistent with the literature [52].

*N-Carbamimidoylfuran-2-carboxamide***2h**. White solid; yield 48%; m.p. 173–175 °C; ^1^H-NMR (300 MHz, DMSO-*d*_6_) δ ppm 6.51 (dd, *J*′ = 1.7 Hz, *J*′′ = 3.3 Hz, 1H), 6.92 (d, *J* = 3.3 Hz, 1H), 7.70 (s, 1H). ^13^C-NMR (62.5 MHz, DMSO-*d*_6_) δ ppm 111.9, 114.0, 144.9, 153.2, 163.2, 168.4; HRMS (ESI) [M − H]^−^ calculated for C_6_H_7_N_3_O_2_: 152.0465, found: 152.0465.

*N,N′-(Iminomethylene)bis(thiophene-2-carboxamide)***2i**. White solid; yield 83%; m.p. 169–171 °C; ^1^H-NMR (300 MHz, DMSO-*d*_6_) δ ppm 7.23 (s, 2H), 7.93 (s, 4H), 9.29 (s, 2H), 12.30 (s, 1H); ^13^C-NMR (62.5 MHz, DMSO-*d*_6_) δ ppm 128.8, 132.1 (2C, overlap), 134.1, 158.9; HRMS (ESI) [M − H]^−^ calculated for C_11_H_9_N_3_O_2_S_2_: 278.0063, found: 278.0063.

*N-(N-Benzoylcarbamimidoyl)-4-methylbenzamide***2j**. Light yellow solid; yield 85%; m.p. 157–159 °C; ^1^H-NMR (300 MHz, CDCl_3_) δ ppm 2.45 (s, 3H), 7.31 (d, *J* = 8.4 Hz, 2H), 7.47–7.61 (m, 3H), 8.00 (d, *J* = 8.4 Hz, 2H), 8.13–8.16 (m, 2H); ^13^C-NMR (62.5 MHz, CDCl_3_) δ ppm 21.7, 128.4, 128.5, 128.6, 129.4, 131.3, 132.7, 135.2, 143.9, 159.2, 173.0, 175.1; HRMS (ESI) [M + H]^+^ calculated for C_16_H_15_N_3_O_2_: 282.1237, found: 282.1238.

*N-(N-Acetylcarbamimidoyl)benzamide***2k**. Yellow solid; yield 43%; m.p. 125–127 °C; ^1^H-NMR (300 MHz, CDCl_3_) δ ppm 2.02 (s, 3H), 7.43–7.57 (m, 3H), 8.16 (m, 2H), 9.34 (bs, 3H); ^13^C-NMR (62.5 MHz, CDCl_3_) δ ppm 30.2, 128.5, 128.9, 132.3, 137.1, 159.5, 163.9, 165.2; HRMS (ESI) [M + H]^+^ calculated for C_10_H_11_N_3_O_2_: 206.0924, found: 206.0952. 

*N-(N-Acetylcarbamimidoyl)-3-methoxybenzamide***2m**. Brown solid; yield 56%; m.p. 87–90 °C; ^1^H-NMR (300 MHz, CDCl_3_) δ ppm 1.88 (s, 3H), 3.83 (s, 3H), 7.10 (dd, 1H, *J*′ = 8.1 Hz, *J*′′ = 2.4 Hz), 7.33–7.38 (m, 1H), 7.72–7.78 (m, 2H), 9.40 (bs, 3H); ^13^C-NMR (62.5 MHz, CDCl_3_) δ ppm 24.4, 55.3, 113.0, 119.2, 121.3, 129.5, 138.4, 159.4, 159.7, 174.1, 178.7; HRMS (ESI) [M + H]^+^ calculated for C_11_H_13_N_3_O_3_: 236.1030 found: 236.1036.

*N,N′-(Iminomethylene)dibenzamide***2n**. White solid; yield 88%; m.p. 159–161 °C; FTIR (Nujol) ν: 3338, 1685, 1653, 1559, 721, 695 cm^−1^; ^1^H-NMR (300 MHz, CDCl_3_) δ ppm 7.50–7.63 (m, 6H), 8.14 (d, *J* = 7.2 Hz, 4H); Spectroscopic data are consistent with the literature [53]. 

*N-(N-Acetylcarbamimidoyl)furan-2-carboxamide***2o**. Light orange solid; yield 67%; m.p. 148–150 °C; ^1^H-NMR (300 MHz, CDCl_3_) δ ppm 2.10 (s, 3H), 6.51 (d, *J* = 1.6 Hz, 1H), 7.19 (d, *J* = 3.3 Hz, 1H), 7.56 (s, 1H); ^13^C-NMR (62.5 MHz, CDCl_3_) δ ppm 29.7, 112.1, 116.7, 145.6, 150.9, 159.1, 169.5, 173.7; HRMS (ESI) [M + H]^+^ calculated for C_8_H_9_N_3_O_3_: 196.0717 found: 196.0755.

*N-(N-Acetylcarbamimidoyl)-2-methylbenzamide***2p**. White solid; yield 47%; m.p. 109–111 °C; ^1^H-NMR (300 MHz, CDCl_3_) δ ppm 1.74 (s, 3H), 2.55 (s, 3H), 7.21–7.25 (m, 2H), 7.35–7.38 (m, 1H), 7.76–7.78 (m, 1H), 9.69 (s, 3H); ^13^C-NMR (62.5 MHz, CDCl_3_) δ ppm 20.9, 24.4, 125.7, 129.2, 130.9, 131.4, 137.1, 138.0, 159.3, 175.2, 180.8; HRMS (ESI) [M + H]^+^ calculated for C_11_H_13_N_3_O_2_: 220.1081 found: 220.1039.

*2-Phenylquinazolin-4-(1H)-one***4a**. White solid; yield 100%; m.p. 238–240 °C; ^1^H-NMR (300 MHz, DMSO-*d*_6_) δ ppm 7.53–7.60 (m, 4H), 7.75 (d, *J* = 8.1 Hz, 1H), 7.83–7.88 (m, 1H), 8.15–8.20 (m, 3H), 12.55 (s, 1H). HRMS (ESI) [M + H]^+^ calculated for (C_14_H_11_N_2_O)^+^: 223.0866, found: 223.0882. Spectroscopic data are consistent with the literature [54].

*1-Methyl-2-phenylquinazolin-4(1H)-one***4b**. White solid; yield 80%; m.p. 160 °C; FTIR (Nujol) ν: 1637, 1606, 1526, 1488, 1256, 1149, 718, 765 cm^−1^; ^1^H-NMR (300 MHz, CDCl_3_) δ ppm 3.71 (s, 3H), 7.47–7.52 (m, 5H), 7.59–7.63 (m, 2H), 7.74–7.79 (m, 1H), 8.35 (dd, *J*′ = 1.4 Hz, *J*′′ = 8.2 Hz, 1H). HRMS (ESI) [M + H]^+^ calculated for (C_15_H_13_N_2_O)^+^: 237.1022, found: 237.1040. Spectroscopic data are consistent with the literature [55].

*2-(4-Fluorophenyl)quinazolin-4(1H)-one***4c**. White solid; yield 87%; m.p. 272–274 °C; ^1^H-NMR (300 MHz, DMSO-*d*_6_) δ ppm 7.38–7.43 (m, 2H), 7.50–7.55 (m, 1H), 7.74 (d, *J* = 8.0 Hz, 1H), 7.82–7.87 (m, 1H), 8.16 (d, *J* = 8.0 Hz, 1H), 8.24–8.28 (m, 2H), 12.59 (s, 1H). HRMS (ESI) [M + H]^+^ calculated for (C_14_H_10_FN_2_O)^+^: 241.0772, found: 241.0788. Spectroscopic data are consistent with the literature [56].

*2-Benzyl-1-methylquinazolin-4(1H)-one***4d**. Yellow solid; yield 75%; m.p. 102–105 °C; FTIR (CH_2_Cl_2_) ν: 1641, 1607, 1548, 1598, 1499, 1265, 1143, 1069, 763, 735, 696 cm^−1^; ^1^H-NMR (300 MHz, CDCl_3_) δ ppm 3.63 (s, 3H), 4.28 (s, 2H), 7.26–7.36 (m, 6H), 7.43–7.49 (m, 1H), 7.67–7.73 (m, 1H), 8.36 (dd, *J*′ = 1.3 Hz, *J*′′ = 7.9 Hz, 1H). HRMS (ESI) [M + H]^+^ calculated for (C_16_H_15_N_2_O)^+^: 251.1179, found: 251.1179. Spectroscopic data are consistent with the literature [57]. 

*2-Benzyl-1-butylquinazolin-4(1H)-one***4e**. White solid; yield 57%; m.p. 89–90 °C; FTIR (Nujol) ν: 1685, 1654, 1560, 1507, 722 cm^−1^; ^1^H-NMR (300 MHz, CDCl_3_) δ ppm 0.91–0.96 (m, 3H), 1.35–1.45 (m, 2H), 1.53–1,58 (m, 2H), 4.02–4.07 (m, 2H), 4.26 (s, 2H), 7.27–7.38 (m, 6H), 7.43–7.48 (m, 1H), 7.68–7.74 (m, 1H), 8.39–8.42 (m, 1H). ^13^C-NMR (62, 5 MHz, CDCl_3_) δ ppm 13.6, 19.9, 30.3, 43.4, 47.0, 114.8, 120.4, 125.9, 127.5, 128.3, 128.9, 129.1, 133.8, 134.8, 140.6, 161,8, 169.0. HRMS (ESI) [M + H]^+^ calculated for (C_19_H_21_N_2_O)^+^: 293.1684, found: 293.1659. 

*2-(4-Methoxyphenyl)-1-methylquinazolin-4(1H)-one***4f**. White solid; yield 64%; m.p. 162–164 °C; FTIR (Nujol) ν: 1648, 1604, 1522, 1507, 1260, 1180, 854, 722, 773 cm^−1^; ^1^H-NMR (300 MHz, CDCl_3_) δ ppm 3.74 (s, 3H), 3.85 (s, 3H), 6.97 (d, *J* = 8.8 Hz, 2H), 7.43–7.48 (m, 2H), 7.60 (d, *J* = 8.8 Hz, 2H), 7.70–7.76 (m, 1H), 8.32 (dd, *J*′ = 1.4 Hz, *J*′′ = 8.0 Hz, 1H). ^13^C-NMR (62.5 MHz, CDCl_3_) δ ppm 38.4, 55.5, 113.9, 115.4, 120.3, 125.9, 126.8, 128.3, 131.1, 133.8, 142.0, 161.6, 162.2, 168.9. HRMS (ESI) [M + H]^+^ calculated for (C_16_H_15_N_2_O_2_)^+^: 267.1128, found: 267.1149.

*1-Ethyl-2-(4-methoxyphenyl)quinazolin-4(1H)-one***4g**. White solid; yield 72%; m.p. 195–196 °C; FTIR (Nujol) ν: 3174, 1675, 1601, 1526, 1289, 1258, 1187, 1032, 848, 768 cm^−1^; ^1^H-NMR (300 MHz, CDCl_3_) δ ppm 1.33–1.37 (m, 3H), 3.90 (s, 3H), 4.23–4.30 (m, 2H), 7.01–7.04 (m, 2H), 7.49–7.56 (m, 4H), 7.75–7.81 (m, 1H), 8.43–8.46 (m, 1H). ^13^C-NMR (62.5 MHz, CDCl_3_) δ ppm 14.3, 43.9, 55.5, 114.0, 115.5, 121.0, 126.0, 127.6, 129.0, 129.7, 133.7, 140.4, 161.1, 162.5, 168.7. HRMS (ESI) [M + H]^+^ calculated for (C_17_H_17_N_2_O_2_)^+^: 281.1285, found: 281.1304.

*2-(4-Methoxyphenyl) quinazolin-4(1H)-one***4h**. Light brown solid; yield 82%; m.p. 237 °C (dec.); ^1^H-NMR (300 MHz, DMSO-*d*_6_) δ ppm 3.86 (s, 3H), 7.10 (d, *J* = 8.9 Hz, 2H), 7.47–7.52 (m, 1H), 7.71 (d, *J* = 7.8 Hz, 1H), 7.79–7.85 (m, 1H), 8.14 (dd, *J*′ = 1.1 Hz, *J*′′ = 7.9 Hz, 1H), 8.20 (d, *J* = 8.9 Hz, 2H), 12.42 (s, 1H). HRMS (ESI) [M + H]^+^ calculated for (C_15_H_13_N_2_O_2_)^+^: 253.0972, found: 253.0994. Spectroscopic data are consistent with the literature [58].

*2-(4-(Trifluoromethyl)phenyl)quinazolin-4(1H)-one***4i**. White solid; yield 58%; m.p. 230 °C (dec.); ^1^H-NMR (300 MHz, DMSO-*d*_6_) δ ppm 7.55–7.60 (m, 1H), 7.78–7.96 (m, 4H), 8.19 (d, *J* = 8.1 Hz, 1H), 8.38 (d, *J* = 8.1 Hz, 2H), 12.76 (s, 1H). HRMS (ESI) [M + H]^+^ calculated for (C_15_H_10_F_3_N_2_O)^+^: 291.0739 found: 291.0770. Spectroscopic data are consistent with those of literature [59].

*2-Benzyl-1-ethylquinazolin-4(1H)-one***4j**. White solid; yield 49%; m.p. 103–104 °C.; ^1^H-NMR (300 MHz, CDCl_3_) δ ppm 1.24 (t, *J* = 7.2 Hz, 3H), 4.18 (q, *J*′ = 7.2 Hz, *J*′′ = 14.1 Hz, 2H), 4.27 (s, 2H), 7.27–7.39 (m, 6H), 7.44–7.49 (m, 1H), 7.68–7.74 (m, 1H), 8.40–8.42 (m, 1H); ^13^C-NMR (62.5 MHz, CDCl_3_) δ ppm 13.5, 42.1, 43.1, 114.7, 125.9, 127.5, 128.4, 129.1, 130.1, 133.9, 134.8, 140.3, 152.9, 161.8, 169.1; HRMS (ESI) [M + H]^+^ calculated for (C_17_H_17_N_2_O)^+^: 265.1335, found: 265.1359.

*2-Benzyl-quinazolin-4(1H)-one***4l**. White solid; yield 95%; m.p. 247 °C; FTIR (Nujol) ν: 3411, 1684, 1617, 1559, 1254, 1162, 889, 718, 775 cm^−1^; ^1^H-NMR (300 MHz, DMSO-*d*_6_) δ ppm 3,94 (s, 2H), 7,25–7,47 (m, 6H), 7.60–7.62 (m, 1H), 7.75–7.77 (m, 1H), 8.07–8.09 (m, 1H), 12.42 (s, 1H); HRMS (ESI) [M + H]^+^ calculated for (C_15_H_12_N_2_O)^+^: 237.1022, found: 237.1041. Spectroscopic data are consistent with those of literature [59].

### 4.3. General Procedure for the Synthesis of 2-(1,2,4 Oxadiazol-5-yl)Benzenamine ***3***

**Method A**: To a solution in ethanol (30mL) of appropriate amidoxime (1 mmol) isatoic anhydride (1 eq) was added. The reaction mixture was heated to reflux for about 5 h and monitored by TLC. The mixture was evaporated, and the oxadiazole was purified by column chromatography using a mixture of petroleum ether and ethyl acetate (20:1) as eluent.

**Method B**: A solution of isatoic anhydride (1 mmol) in DMSO (20 mL) was treated with K_2_CO_3_ (1 mmol) and the appropriate alkyl halide (1.1 mmol) and stirred at room temperature for 12 h. The reaction was monitored by TLC. The solvent was evaporated under a vacuum, and the residue was treated with water and extracted with ethyl acetate. The organic layer was dried with Na_2_SO_4_ and evaporated under a vacuum. The solid, without further purification, was solubilized in toluene and heated to reflux with the amidoxime (1.5 eq). The reaction was monitored with TLC for about 3 h, and then the solvent was removed under vacuum. The desired product was isolated by chromatography using a mixture of petroleum ether and ethyl acetate (20:1) as eluent. 

**Method C**: 5-(2-fluorophenyl)-1,2,4-oxadiazole (1 mmol) [60] was reacted with methylamine or ethylamine (10 mmol) in EtOH (10 mL) into a pressure tube. The tube was stirred in an oil bath at the temperature of 80 °C for 12 h monitoring the reaction by TLC. The desired product was isolated by column chromatography using a mixture of petroleum ether and ethyl acetate (20:1) as eluent. 

*2-(3-Phenyl-1,2,4 oxadiazol-5-yl)benzenamine***3a** (Method A). White solid; yield 58%; m.p. 128–129 °C; ^1^H-NMR (300 MHz, DMSO-*d*_6_) δ ppm 6.68–6.73 (m, 1H), 6.95–7.00 (m, 3H), 7.33–7.39 (m, 1H), 7.60–7.63 (m, 3H), 7.86 (dd, 1H, *J*′ =1.3 Hz, *J*′′ = 8.0 Hz), 8.14–8.17 (m, 2H); ^13^C-NMR (62.5 MHz, DMSO-*d*_6_) δ ppm 104.0, 116.1, 117.0, 126.7, 127.7, 128.9, 129.6, 131.9, 134.5, 149.3, 167.7, 175.2; HRMS (ESI) [M + H]^+^ calculated for (C_14_H_12_N_3_O)^+^: 238.0975, found: 238.0975.

*2-(3-Phenyl-1,2,4-oxadiazol-5-yl)-N-methylbenzenamine***3b** (Method B, C). White solid; yield 35–55%; m.p. 78–80 °C; FTIR (Nujol) ν: 3361, 1617, 1583, 1544, 1526, 1253, 1139, 742, 720 cm^−1^; ^1^H-NMR (300 MHz, DMSO-*d*_6_) δ ppm 3.02 (d, 3H, *J* = 4.9 Hz), 6.74–6.82 (m, 2H), 7.44–7.50 (m, 1H), 7.54–7.56 (m, 3H), 7.8 (s, 1H), 8.05 (dd, 1H, *J*′ = 1.2 Hz, *J*′′ = 7.8 Hz), 8.17–8.20 (m, 2H); ^13^C-NMR (62.5 MHz, DMSO-*d*_6_) δ ppm 29.9, 105.9, 110.9, 115.4, 127.0, 127.5, 128.8, 129.5, 131.2, 134.3, 149.0, 167.7, 175.0; HRMS (ESI) [M + H]^+^ calculated for (C_15_H_14_N_3_O)^+^: 252.1131, found: 252.1131.

*2-[3-(4-Fluoropenyl)-1,2,4-oxadiazol-5-yl]benzenamine***3c** (Method A). White solid; yield 48%; m.p. 126–129 °C; ^1^H-NMR (300 MHz, DMSO-*d*_6_) δ ppm 6.67–6.72 (m, 1H), 6.95–7.00 (m, 3H), 7.33–7.38 (m, 1H), 7.41–7.47 (m, 2H), 7.85 (dd, 1H, *J*′ = 1.2 Hz, *J*′′ = 8.1 Hz), 8.23 (dd, 2H, *J*′ = 5.4 Hz, *J*′′ = 8.7 Hz); ^13^C-NMR (62.5 MHz, DMSO-*d*_6_) δ ppm 103.9, 116.1, 116.6, 117.0 (d, *J* = 11.3 Hz), 123.3 (d, *J* = 3 Hz), 128.9, 130.2 (d, *J* = 8.9 Hz), 134.5, 149.3, 164.4 (d, *J* = 247.7 Hz), 166.9, 175.2.; HRMS (ESI) [M + H]^+^ calculated for (C_14_H_11_FN_3_O)^+^: 256.0881 found: 256.0884.

*2-(3-Benzyl-1,2,4-oxadiazol-5-yl)-N-methylbenzenamine***3d** (Method B, C). White solid; yield 46–61%; m.p. 57 °C; FTIR (Nujol) ν: 3352, 1617, 1559, 1258, 1174, 743, 726 cm^−1^; ^1^H-NMR (300 MHz, DMSO-*d*_6_) δ ppm 2.92 (d, 3H, *J* = 4.9 Hz), 4.19 (s, 2H), 6.70–6.75 (m, 1H), 6.84 (d, 1H, *J* = 8.6 Hz), 7.25–7.39 (m, 4H), 7.45–7.50 (m, 2H), 7.86 (d, 1H, *J* = 8.0 Hz). ^13^C-NMR (62.5 MHz, DMSO-*d*_6_) δ ppm 29.9, 31.9, 104.7, 111.7, 115.7, 127.4, 129.0, 129.3, 129.4, 135.1, 136.3, 148.9, 169.1, 174.7. HRMS (ESI) [M + H]^+^ calculated for (C_16_H_16_N_3_O)^+^: 266.1287, found: 266.1308.

*2-(3-Benzyl-1,2,4-oxadiazol-5-yl)-N-butylbenzenamine***3e** (Method B). Oil; yield 52%; ^1^H-NMR (300 MHz, DMSO-*d*_6_) δ ppm 0.97–1.02 (m, 3H), 1.43–1.50 (m, 2H), 1.66–1.71 (m, 2H), 3.20–3.26 (m, 2H), 4.15 (s, 2H), 6.67–6.76 (m, 1H), 6.84 (d, 1H, *J* = 8.5 Hz), 7.28–7.45 (m, 6H), 7.55–7.57 (m, 1H), 7.84 (d, 1H, *J* = 8.0 Hz). ^13^C-NMR (62.5 MHz, DMSO-*d*_6_) δ ppm 13.9, 20.3, 31.1, 32.5, 42.7, 105.1, 111.2, 115.0, 127.1, 128.6, 129.1, 129.4, 134.2, 135.7, 148.2, 168.7, 175.0. HRMS (ESI) [M + H]^+^ calculated for (C_19_H_22_N_3_O)^+^: 308.1757 found: 308.1791.

*2-[3-(4-Methoxyphenyl)-1,2,4-oxadiazol-5-yl]-N-methylbenzenamine***3f** (Method B). White solid; yield 53%; m.p. 97–99 °C; ^1^H-NMR (300 MHz, DMSO-*d*_6_) δ ppm 3.01 (d, 3H, *J* = 4.7 Hz), 3.86 (s, 3H), 6.73–6.78 (m, 1H), 6.89 (d, 1H, *J* = 8.5Hz), 7.14 (d, 2H, *J* = 8.7 Hz), 7.48–7.56 nv (m, 1H), 7.69–7.70 (m, 1H), 7.91–7.94 (m, 1H), 8.13 (d, 2H, *J* = 8.7 Hz). ^13^C-NMR (62.5 MHz, DMSO-*d*_6_) δ ppm 30.1, 55.9, 104.7, 105.9, 111.8, 115.0, 115.7, 118.8, 129.4, 129.5, 135.1, 149.0, 162.3, 174.6; HRMS (ESI) [M + H]^+^ calculated for (C_16_H_16_N_3_O_2_)^+^: 282.1237, found: 282.1261.

*2-[3-(4-Methoxyphenyl)-1,2,4-oxadiazol-5-yl]-N-ethylbenzenamine***3g** (Method B). White solid; yield 62%; m.p. 98–101 °C; ^1^H-NMR (300 MHz, CDCl_3_) δ ppm 1.43–1.48 (m, 3H), 3.33–3.39 (m, 2H), 3.88 (s, 3H), 6.75 (dd, 2H, *J*′ = 8.2 Hz, *J*′′ = 17.3 Hz), 7.03 (d, 2H, *J* = 8.5 Hz), 7.38–7.43 (m, 1H), 7,75 (s, 1H), 8.04 (dd, 3H, *J*′ = 8.3 Hz, *J*’’ = 14.8 Hz). ^13^C-NMR (62.5 MHz, CDCl_3_) δ ppm 14.6, 37.7, 55.4, 104.6, 105.2, 111.3, 114.2, 115.2, 119.4, 128.9, 129.4, 134.1, 148.1, 161.9, 167.2, 174.7; HRMS (ESI) [M + H]^+^ calculated for (C_17_H_18_N_3_O_2_)^+^: 296.1394, found: 296.1406.

*2-[3-(4-Methoxyphenyl)-1,2,4-oxadiazol-5-yl]-N-benzenamine***3h** (Method A). ^1^H-NMR (300 MHz, DMSO-*d*_6_) 3.86 (s, 3H), 6.67–6.72 (m, 1H), 6.94–6.99 (m, 3H), 7.14 (d, 2H, *J* = 9 Hz), 7.32–7.38 (m, 1H), 7.82–7.85 (m, 1H), 8-07-8.11 (m, 2H); ^13^C-NMR (62.5 MHz, DMSO-*d*_6_) δ ppm 55.8, 104.1, 105.7, 115.0, 116.1, 117.0, 119.0, 128.9, 129.4, 134.4, 149.2, 162.2, 167.4, 174.9; HRMS (ESI) [M + H]^+^ calculated for (C_15_H_14_N_3_O_2_)^+^: 268.1081, found: 268.1094.

*2-[3-[4-(Trifluoromethyl)phenyl]-1,2,4-oxadiazol-5-yl]benzenamine***3i** (Method A). Light brown solid; yield 56%; m.p. 135–138 °C; ^1^H-NMR (300 MHz, DMSO-*d*_6_) δ ppm 6.68–6.73 (m, 1H), 6.97 (d, 1H, *J* = 9 Hz); 7.03 (s, 2H), 7.34–7.40 (m, 1H), 7.87 (dd, 1H, *J*’ = 1.4 Hz, *J*’’ = 8.1 Hz), 7.98 (d, 2H, *J* = 8.2 Hz), 8.39 (d, 2H, *J* = 8.0 Hz); ^13^C-NMR (62.5 MHz, DMSO-*d*_6_) δ ppm 103.7, 116.1, 117.1, 124.3 (q, *J* = 270.8 Hz), 126.5 (q, *J* = 1.25 Hz), 128.5, 129.7, 130.5, 131.8 (q, *J* = 31.8 Hz), 134.6, 149.4, 166.7, 175.5; HRMS (ESI) [M + H]^+^ calculated for (C_15_H_11_F_3_N_3_O)^+^: 306.0849 found: 306.0881.

*2-(3-Benzyl-1,2,4-oxadiazol-5-yl)-N-ethylbenzenamine***3j** (Method B, C). Yellow solid; yield 10–46%; m.p. 60 °C.; ^1^H-NMR (300 MHz, CDCl_3_) δ ppm 1.32–1.37 (m, 3H), 3.23–3.32 (m, 2H), 4.15 (s, 2H), 6.67–6.75 (m, 2H), 7.28–7.42 (m, 7H), 7.56 (s, 1H), 7.93–7.96 (dd, *J*’ = 1.2 Hz *J*’’ = 7.8 Hz, 1H); ^13^C-NMR (62.5 MHz, CDCl_3_) δ 14.7, 31.8, 37.5, 104.4, 112.1, 115.8, 127.4, 129.0, 129.3, 129.5, 135.1, 136.2, 148.1, 169.1, 174.6; HRMS (ESI) [M + H]^+^ calculated for (C_17_H_18_N_3_O)^+^: 280.1444 found: 280.1468.

*2-[3-(4-Chlorophenyl)-1,2,4-oxadiazol-5-yl]benzenamine***3k** (Method A). White solid; yield 44%; m.p. 141–144 °C; ^1^H-NMR (300 MHz, CDCl_3_) δ ppm 5.98 (s, 2H), 6.80–6.86 (m, 2H), 7.35–7.40 (m, 1H), 7.52 (d, 2H, *J* = 8.4 Hz), 8.02 (d, 1H, *J* = 8.7 Hz), 8,12 (d, 2H, *J* = 8.4 Hz). ^13^C-NMR (62.5 MHz, CDCl_3_) δ ppm 103.8, 116.1, 117.1, 125.6, 128.9, 129.5, 129.8, 134.6, 136.8, 149.4, 167.0, 175.3. HRMS (ESI) [M + H]^+^ calculated for (C_14_H_11_ClN_3_O)^+^: 272.0585, found: 272.0623.

*2-(3-Benzyl-1,2,4-oxadiazol-5-yl)benzenamine***3l** (Method A). Light brown solid; yield 49%; m.p. 87–89 °C; ^1^H-NMR (300 MHz, DMSO-*d*_6_) δ ppm 4.17 (s, 2H), 6.64–6.67 (m, 1H), 6.82 (s, 2H), 6.88 (d, 1H, *J* = 8.4 Hz), 7.28–7.37 (m, 6H), 7.80 (d, 1H, *J* = 8.0 Hz). ^13^C-NMR (62.5 MHz, DMSO-*d*_6_) δ ppm 31.9, 104.1, 116.1, 116.9, 127.3, 128.8, 129.0, 129.4, 134.4, 136.3, 149.1, 169.2, 174.9. HRMS (ESI) [M + H]^+^ calculated for (C_15_H_14_N_3_O)^+^: 252.1131, found: 252.113. 

### 4.4. General Procedure for the Synthesis of N-Benzyl-2-(3-Phenyl-1,2,4-Oxadiazol-5-yl)Benzenamine ***3m***

A solution of the 1,2,4-oxadiazole **3a** (1 mmol) in CH_2_Cl_2_ (10 mL) reacted with benzaldehyde (1.1 mmol) in the presence of sodium triacetoxyborohydride (3 mmol) and acetic acid (2.2 mmol). The reaction was stirred at room temperature and monitored by TLC. After 24 h, NaOH 1 M (3 mL) was added to the mix, and the solvent was evaporated under vacuum. The residue was treated with water and extracted with chloroform (CHCl_3_). The organic layer was dried with Na_2_SO_4_ and evaporated under a vacuum. The product was isolated by column chromatography using a mixture of petroleum ether and ethyl acetate (5:1) as eluent. 

*N-Benzyl-2-(3-phenyl-1,2,4-oxadiazol-5-yl)benzenamine***3m**. Yellow solid; yield 81%; m.p. 132–134 °C; ^1^H-NMR (300 MHz, CDCl_3_) δ ppm 4.60 (d, 2H, *J* = 5.3 Hz), 6.77–6.82 (m, 2H), 7.35–7.54 (m, 8H), 8.04–8.09 (m, 3H), 8.41 (m, 1H); ^13^C-NMR (62.5 MHz, CDCl_3_) δ ppm 47.4, 105.7, 111.8, 115.9, 126.9, 127.2, 127.4, 127.5, 128.8 (2C, overlap), 129.5, 131.2, 134.4, 138.7, 148.0, 167.6, 174.9; HRMS (ESI) [M + H]^+^ calculated for (C_21_H_18_N_3_O)^+^: 328.1444, found: 328.1452.

### 4.5. DPPIV Activity Assay

The DPPIV activity assay was performed using DPPIV Activity Fluorometric Assay Kit (Sigma Aldrich, St. Louis, MO, USA) in accordance with the manufacturer’s instructions. The compounds dissolved in DMSO were diluted to initial concentration of 10 mM and tested at 100 µM. The enzyme inhibition test was conducted in a 96-well flat for fluorescence assay. To the wells have been added 25 µL of tested compound (400 µM), 49 µL of assay buff.

After, an enzymatic reaction mix (23 µL of assay buffer and 2 µL of substrate) was added to each well. The DPPIV activity was determined by cleaving the substrate to yield a fluorescence product (λ_ex_ = 360/λ_em_ = 460) proportional to the enzymatic activity. The measure of fluorescence was made in kinetic mode for 30 min at 37 °C. The fluorescence values were taken every minute. Sitagliptin was used as a positive control. The fluorescence was plotted against time, and the enzyme inhibition percentage was calculated as follows:

% Relative Inhibition = (Slope EC-Slope SM)/Slope EC × 100.

Slope SM = the slope of the compound’s curve.

Slope EC = the slope of the Enzyme Control curve.

The IC_50_ values have been calculated evaluating the inhibition percentage in the presence of 100, 10, 1, 0.1 and 0.01 µM of the compounds.

### 4.6. α-Glucosidase Inhibition Test

An *α*-Glucosidase inhibition assay was performed according to the method previously reported in the literature [61]. The compounds were prepared in DMSO at a concentration of 10 mM and were tested at five concentrations (100, 10, 1, 0.1 and 0.01 µM). The inhibition test was conducted in a 96-well flat. In each well, we added 70 µL of Na_2_HPO_4_ buffer (50 mM) pH 6.8, 10 µL of the tested compounds and 10 µL of enzyme solution in buffer (0.0234 U). The plate was pre-incubated for 10 min at 37 °C and pre-read at 400 nm in absorbance, using a 96-well microplate reader (BioTek, Winooski, VT, USA). The substrate p-nitrophenyl glucopyranoside was added and the plate was incubated at 37 °C for another 30 min. The absorbance, due to the formation of p-nitrophenol, was measured at 400 nm. Acarbose was used as a positive control. The percentage of inhibition was found as follows: % inhibition = abs of control − abs of test compound/abs of control × 100.

### 4.7. MTS Assay

The MTS assay (Promega Italia, S.r.l., Milan, Italy) was performed in accordance with the manufacturer’s instructions. The cells were plated on a 96-well plate up to a concentration of ≈6 × 10^5^ cell/mL. After 24 h, the cells were treated with the compounds at the concentration of 12.5, 25, 50 and 100 µM. A solution of MTS [3-(4,5-dimethylthiazol-2-yl)-5-(3-carboxymethoxyphenyl)-2-(4-sulfophenyl)-2*H*-tetrazolium] (20 µL) was added to each well and the plate was incubated for 4 h at 37 °C under atmosphere of CO_2_/5%. The absorbance at 490 nm was read with a microplate reader WallacVictor 2 1420 Multilabel Counter (PerkinElmer, Monza, Italy). The results are reported as the absorbance values of treated cells compared to those of the control: % cell viability = (OD compound/OD Control) × 100.

### 4.8. Analysis of Reactive Oxygen Species (ROS) 

The cells were plated on a 96-well plate up to a concentration of 6 × 10^5^ cell/mL and were treated with the compounds at the concentration of 50 µM and 100 µM. After the treatment, a 1 mM solution of DCFH-DA in PBS was added to the wells. The plate was incubated for 10 min at room temperature and away from light. The cells were washed with PBS and the fluorescence (λ_ex_ = 485 nm/λ_em_ = 530 nm) was monitored by a microplate reader GloMax fluorimeter (Promega Corporation, Madison, USA) and a fluorescence microscope Zeiss Axio Scope 2 (Zeiss, Jena, Germany)

## 5. Conclusions

This study highlights an ammonium formate-Pd/C system as a valid reducing agent for O-N bond of 1,2,4-oxadiazoles, allowing us to obtain amidines, guanidines and other derivatives. This strategy also obtained quinazolinones through a new reduction-induced ring-rearrangement of *ortho*-arylamino-oxadiazole compounds. This strategy make available the alkaloid Glicosine, here identified as an effective DPPIV inhibitor, with potential applications as an antidiabetic drug also in light of the good cytocompatibility and the lack of induction of oxidative stress. Glicosine is a promising lead compound that is selective toward DPPIV and will be further optimized in the future. 

## Figures and Tables

**Table 1:** Optimization of reduction reaction of 1,2,4-oxadiazole **1a**.

Entry	Solvent	Reducing Agent	Catalyst	T (°C)	Time	2a Yield %
1	THF	NH_4_CO_2_H	Pd/C (5%)	25 °C	48 h	NR ^1^
2	Toluene	NH_4_CO_2_H	Pd/C (5%)	25 °C	48 h	NR ^1^
3	AcOEt	NH_4_CO_2_H	Pd/C (5%)	25 °C	48 h	NR ^1^
4	CHCl_3_	NH_4_CO_2_H	Pd/C (5%)	25 °C	48 h	NR ^1^
5	CH_3_CN	NH_4_CO_2_H	Pd/C (5%)	25 °C	48 h	NR ^1^
6	MeOH	NH_4_CO_2_H	none	25 °C	48 h	NR ^1^
7	MeOH	NH_4_CO_2_H	Zn	25 °C	48 h	NR ^1^
8	MeOH	NH_4_CO_2_H	Pd/C (5%)	25 °C	48 h	15% ^2^
9	EtOH	NH_4_CO_2_H	Pd/C (5%)	25 °C	48 h	10% ^2^
10	MeOH	HCO_2_H	Pd/C (5%)	25 °C	48 h	13% ^2^
11	MeOH	HCO_2_H	Pd/C (5%)	60 °C	1 h	78% ^2^
12	MeOH	NH_4_CO_2_H	Pd/C (5%)	60 °C	1 h	90% ^2^

^1^ NR: No reaction. ^2^ Isolated yields.

**Table 2:** DPPIV inhibitory activity of the tested compounds at 100 µM and IC_50_ values obtained in DPPIV assay by fitting the percent enzymatic activity after treatment with the indicated compounds at 0.01, 0.1, 1, 10 and 100 µM.

Compound	Inhibition% (100 µM)	IC_50_ (µM)
**2e**	37.3	ND ^1^
**2k**	79.3	ND ^1^
**2n**	82.7	ND ^1^
**4b**	83.2	0.157
**4d**	83.9	0.038
**4e**	87.1	0.619
**4f**	82.4	ND ^1^
**4h**	79.1	ND ^1^
**4i**	72.5	ND ^1^
**4l**	98.0	0.436
**Sitagliptin**	92.1	0.026

^1^ ND: not determined.
